# Changes in home visit utilization during the COVID-19 pandemic: a multicenter cross-sectional web-based survey

**DOI:** 10.1186/s13104-022-06128-7

**Published:** 2022-07-07

**Authors:** Jun Hamano, Hirokazu Tachikawa, Sho Takahashi, Saori Ekoyama, Hiroka Nagaoka, Sachiko Ozone, Shoichi Masumoto, Takahiro Hosoi, Tetsuaki Arai

**Affiliations:** 1 Division of Clinical Medicine, Faculty of Medicine, University of Tsukuba, 1-1-1 Tennoudai, Tsukuba, Ibaraki 305-8575 Japan; 2 Department of Disaster and Community Psychiatry, Faculty of Medicine, University of Tsukuba, Tsukuba, Japan; 3 University of Tsukuba Hospital, Tsukuba, Japan; 4 Department of Family Medicine, General Practice and Community Health, Faculty of Medicine, University of Tsukuba, Tsukuba, Japan; 5 Department of General Medicine, Tsukuba Central Hospital, Kamikashiwada 4-58-1, Ushiku, Ibaraki 300-1232 Japan; 6 Division of Clinical Medicine, Department of Psychiatry, Faculty of Medicine, University of Tsukuba, 1-1-1 Tennoudai, Tsukuba, Ibaraki 305-8575 Japan

**Keywords:** Home visits, COVID-19 pandemic, Multicenter study, Cross-sectional study

## Abstract

**Objective:**

Home care is one of the essential community health care services; thus, identifying changes of home care utilization before and during the COVID-19 pandemic would be useful for researchers and policymaker to reconsider the home care system, the support needed for home care staff, and the collaborative system with hospitals in the COVID-19 era. We conducted a multicenter cross-sectional web-based anonymous survey of the directors of home visit facilities in Japan in August 2021.

**Results:**

A total of 33 participants from 37 facilities responded to the survey. The number of patients dying at home and newly requested home visits increased during the COVID-19 pandemic (74.2%, 71.0%). One possible reason was the restricted visitation of inpatient facilities (93.5%). The underlying disease that the largest number of participants perceived as having increased compared with before the COVID-19 pandemic was cancer (51.6%). There were no significant differences in being in a rural area or the number of doctors in perceived changes in home visit utilization. Our study indicated that the director of home visit facilities thought the number of patients dying at home and newly requested home visits had increased compared with before the COVID-19 pandemic.

**Supplementary Information:**

The online version contains supplementary material available at 10.1186/s13104-022-06128-7.

## Introduction

The COVID-19 pandemic drastically changed health care utilization for health-related events worldwide [1–7]. The major reasons for these changes were patients avoiding in-person health care due to the fear of exposure to COVID-19, and governments’ implementation of various strategies such as large-scale physical distancing measures and movement restrictions [8].

Several previous studies revealed changes in health care utilization in the outpatient clinic, hospital setting, and home care service due to the COVID-19 pandemic [4, 5, 9–11]. Xu et al. reported significant reductions in inpatients, emergency department, and outpatient utilization in the early days of the pandemic [5]; however, other studies reported that the evidence in the home care setting in the USA was controversial [11, 12]. Chou et al. noted no significant difference in new enrollments in home hospices; however, Karen et al. reported an increase in the admission rate to home hospices. Moreover, Aoki et al. reported that the number of visits to lower-level medical institutions, such as physicians’ office and hospital outpatient clinics, decreased remarkably compared to pre-pandemic levels. However, the change of the number of home care uses was unclear in Japan.

As home care is one of the essential community health care services, identifying changes of home care utilization before and during the COVID-19 pandemic would be useful for researchers and policymakers to reconsider the home care system, the support needed for home care staff, and the collaborative system with hospitals in the COVID-19 era. In the present study, we investigated changes in the home health care utilization during the COVID-19 pandemic.

## Main text

### Methods

#### Study design and setting

A multicenter cross-sectional web-based anonymous survey of home health care workers was conducted in August 2021, during the fifth wave of the pandemic in Japan, using the SurveyMonkey platform [13]. SurveyMonkey is an online service that facilitates sharing surveys via email, smartphone applications, and social media platforms such as Facebook and Twitter. We sent the URL of the survey via e-mail to each participant in August 2021, and sent a reminder e-mail to all participants 3 weeks after sending the first e-mail.

This study was conducted under the ethical standards of the Declaration of Helsinki and the ethical guidelines for epidemiological research issued by the Ministry of Health, Labour and Welfare of Japan. The Institutional Review Board of the University of Tsukuba approved this study (No. 1651).

#### Participants

We recruited facilities that provide home visit services using the primary care research networks, which explore the distress of end-of-life patients at home [14–16]. The inclusion criteria were: (1) provide home visit services during the COVID-19 pandemic, and (2) the director of the facility had given prior consent to participate in the study. Exclusion criteria were: (1) duplicate responses from the same IP address, and (2) no response. We explained the purpose of the web-based anonymous survey and asked for consent to participate in this study using a web page.

#### Questionnaire

In the absence of specific and validated instruments for evaluating perceived changes in home visit utilization and possible reasons during the COVID-19 pandemic, we developed an original questionnaire based on previous studies and discussion among the authors of the present study [5, 9–11]. We confirmed the content validity by three physicians, and face validity of the questionnaires by three psychiatrists and one social worker. We asked the respondents to respond to the statements including the following: “Compared with before the COVID-19 pandemic, the number of newly requested home visits has increased: yes/no”, “Compared with before the COVID-19 pandemic, the number of home visits patients with a short prognosis has increased: yes/no”, “Compared with before the COVID-19 pandemic, the number of patients dying at home has increased: yes/no”, “Many patients and their families requested home visits after being recommended by the physician or nurse at a hospital: yes/no”, “Many patients and their families wanted to stay at home because they were worried about being infected with COVID-19 at a hospital: yes/no”, and “Many patients and families wanted home visits because the inpatient facilities were restricting visitation: yes/no”. In addition, we asked about the primary diseases in newly requested home visits that increased in incidence compared with before the COVID-19 pandemic (Additional file 1: Appendix).

#### Statistical analysis

Descriptive statistics were calculated for the perceived changes in home visit utilization and possible reasons. As previous studies indicated that being in a rural area and the number of physicians were candidate factors that were associated with the management of home care patients and the impact of COVID-19 pandemic on their practice [17, 18], we performed chi-square test to explore the association of being in a rural area (the population of the municipality where the facilities are located was defined as rural if it was less than 100,000) and solo practice (the facility had only one full-time physician). All analyses were carried out using SPSS-J software (ver. 27.0; IBM, Tokyo, Japan).

### Results

A total of 33 participants from 37 facilities responded to the survey. We excluded two participants whose responses could not be registered. Fourteen participants were in a rural area and nine participants were in solo practice.

The most frequently perceived change in home visit utilization was “the number of patients dying at home has increased” (74.2%), followed by “the number of newly requested home visits has increased” (71.0%). The most frequent reason for the change in home visit utilization was “the inpatient facilities were restricting visitation” (93.5%) (Table 1).

**Table 1:** The perceived changes in home visit utilization and the possible reasons

	All (n = 31)		Location					Number of doctors				
	Yes	%	Rural (n = 14)	%	Urban (n = 17)	%	p	Solo practice (n = 9)	%	Two or more doctors (n = 22)	%	p
Compared with before the COVID-19 pandemic												
The number of newly requested home visits has increased	22	71.0	10	71.4	12	70.6	0.959	6	66.7	16	72.7	0.736
The number of home visit patients with a short prognosis has increased	12	38.7	4	28.6	8	47.1	0.293	3	33.3	9	40.9	0.694
The number of patients dying at home has increased	23	74.2	10	71.4	13	76.5	0.750	6	66.7	17	77.3	0.540
Many patients and their families												
Requested home visits after being recommended by a physician or nurse at a hospital	17	54.8	9	64.3	8	47.1	0.337	4	44.4	13	59.1	0.457
Wanted to stay at home because they were worried about being infected with COVID-19 at a hospital	13	41.9	5	35.7	8	47.1	0.717	4	44.4	9	40.9	0.856
Wanted home visits because the inpatient facilities were restricting visitation	29	93.5	13	92.9	16	94.1	0.887	8	88.9	21	95.5	0.499

The underlying disease that the largest number of participants perceived as having increased compared with before the COVID-19 pandemic was cancer (51.6%), followed by respiratory disease (25.8%) and dementia (22.6%). There were no significant differences in being in a rural area or the number of doctors in perceived changes in home visit utilization (Table 2).

**Table 2:** The underlying diseases of patients with newly requested home visits thought to have increased in incidence compared with before the COVID-19 pandemic

	All		Location					Number of doctors				
	Yes (n = 31)	%	Rural (n = 14)	%	Urban (n = 17)	%	p	Solo practice (n = 9)	%	Two or more doctors (n = 22)	%	p
Cardiovascular disease	5	16.1	2	14.3	3	17.6	0.800	1	11.1	4	18.2	0.627
Cerebrovascular disease	3	9.7	1	7.1	2	11.8	0.665	1	11.1	2	9.1	0.863
Respiratory disease	8	25.8	3	21.4	5	29.4	0.613	2	22.2	6	27.3	0.771
Liver disease	2	6.5	1	7.1	1	5.9	0.887	1	11.1	1	4.5	0.499
Renal disease	1	3.2	1	7.1	0	0.0	0.263	1	11.1	0	0.0	0.112
Neurological disease	3	9.7	1	7.1	2	11.8	0.665	1	11.1	2	9.1	0.863
Musculoskeletal disorders	1	3.2	1	7.1	0	0.0	0.263	1	11.1	0	0.0	0.112
Mental disorder	2	6.5	2	14.3	0	0.0	0.107	1	11.1	1	4.5	0.499
Dementia	7	22.6	2	14.3	5	29.4	0.316	2	22.2	5	22.7	0.976
Cancer	16	51.6	8	57.1	8	47.1	0.576	5	55.6	11	50.0	0.779

### Discussion

To the best of our knowledge, this is the first nationwide survey to explore the perceived changes in home visit utilization during the COVID-19 pandemic. A notable finding of our study was that the directors of home visit facilities thought that the number of patients dying at home and newly requested home visits, especially for cancer patients, increased compared with before the COVID-19 pandemic. This is inconsistent with the result of a cohort study in Taiwan that indicated there was no significant change in the trend of hospice home care utilization and new enrollments in the hospice home care program before and during the COVID-19 pandemic [11]. One possible reason was the increase in the threshold for hospital utilization due to fear of being infected by COVID-19 at hospital, which was reported in a previous study in Japan [1]. This phenomenon also matches the findings of a previous study that reported that Japanese people tended to be more risk-averse than other nationalities [19]. Another possible reason was that the number of beds in hospitals, especially in palliative care units, was decreased to take care of COVID-19 patients [20]. Thus, our study suggested that more patients, especially cancer patients, used home visits and stayed at home until death than before the COVID-19 pandemic. This likely led to an increase in the physical and phycological burden on home care staff. Therefore, it is important to implement a support system for home care staff such as the mutual support system among home care facilities that was adopted in Taiwan [21].

Another notable finding of this study was that most directors of home visit facilities thought that restricted visitation of inpatient facilities led to an increase in home visit utilization. A previous review reported that if visitation restrictions had a negative impact on mental health outcome of patients and their families, the patient and family preferred to stay at or go back to their home [22]. This would affect decisions about health care utilization not only by the patient and their family, but also by health care professionals. Thus, we need to compare the patients’ outcome of health care utilization before and during the COVID-19 pandemic by different research methods such as claims data survey or bereaved family survey.

Of note, there were no significant differences in being in a rural area or the practice style (solo practice or not) in perceived changes in home visit utilization. This finding suggests that changes in home visit utilization during the COVID-19 pandemic were not influenced by the amount of local health care resources. It is possible that some areas were overburdened with home health care workers. Thus, some practical mutual support systems; such as human support from other home care facilities or hospitals, and mental health support from experts in the community, would be needed in the COVID-19 era [21].

### Conclusion

Our study indicated that the directors of home visit facilities thought that the number of patients dying at home and newly requested home visits had increased compared with before the COVID-19 pandemic. Moreover, most directors of home visit facilities thought that the restricted visitation of inpatient facilities led to an increase in home visit utilization.

## Limitations

- The data were based on the perception of the directors of home visit facilities. Thus, it was difficult to assure objectivity and generalizability. Future studies based on claims data or medical records are needed.
- As we designed this study as exploratory research, we did not estimate the sample size before conducting the survey; therefore, the number of participants was not feasible to assess statistical significance. However, our data may contribute to the improvement of home health care in the COVID-19 era.
- We used variables that have not been validated. However, we believe that these limitations did not have a significant impact on our study, which revealed an important issue that needs to be addressed in the COVID-19 era.

## Supplementary Information

**Additional file 1: Appendix** Questionnaire for the director of the facility.

## Data Availability

The datasets generated during and analyzed during the present study are not publicly available due to them containing information that could compromise research participant privacy/consent; however, they are available from the corresponding author on reasonable request.
