# Supplementary material for: Changes in home visit utilization during the COVID-19 pandemic: a multicenter cross-sectional web-based survey
Source: BMC Res Notes. 2022 Jul 7;15:238. doi: 10.1186/s13104-022-06128-7 (PMC9261221; doi:10.1186/s13104-022-06128-7)
Supplement: Supplementary file 1 — Additional file 1: Appendix Questionnaire for the director of the facility. [file 13104_2022_6128_MOESM1_ESM.pdf]

Questionnaire for the director of the facility

1. Multiple cases of novel coronavirus infection at collaborating medical institutions and facilities in the past month.

Yes ・ No

2. More people have retired or wish to retire compared to before the novel coronavirus outbreak.

Yes ・ No

3. Many patients and families requested home visits because the inpatient facilities were restricting visitation.

Yes ・ No

4. Many patients/families wanted to go home because they were worried about contracting COVID-19 in hospital.

Yes ・ No

5. Many patients/families wanted to go home after being recommended by doctors and nurses.

Yes ・ No

6. Compared with before the COVID-19 pandemic, the number of newly requested patients with shorter prognosis at home has increased.

Yes ・ No

7. Compared with before the COVID-19 pandemic, the number of dying patients at home has increased.

Yes ・ No

8. Compared with before the COVID-19 pandemic, the number of newly requested home visits has increased.

Yes ・ No

If you answered "yes," please answer the following questions

9. What was the biggest increase in the primary disease of newly requested homebound patients compared to before the new coronavirus pandemic?

Cancer ・Dementia ・Heart failure ・Respiratory failure ・Failure to thrive ・Other (free description)
